# Proposed Features for a Digital Nutrition Intervention for Managing Parkinson’s Disease

**DOI:** 10.1093/geroni/igaa057.824

**Published:** 2020-12-16

**Authors:** Dara LoBuono, Sarah Dobiszewski, Alison Tovar, Skye Leedahl, Furong Xu, Matthew Delmonico, Leslie Mahler, Ingrid Lofgren

**Affiliations:** 1 University of Rhode Island, North Haledon, New Jersey, United States; 2 University of Rhode Island, Kingston, Rhode Island, United States

## Abstract

Digital health technologies can enhance quality of care for people with Parkinson’s disease (PwPD) and their informal caregivers (ICG), but research in this area is lacking. Developing digital health nutrition services incorporating end-user preferences is essential. This cross-sectional, mixed-method study assessed 20 PwPD and their ICG. Participants reported sources they first search for when seeking health information, the amount of effort it takes to find the information, their confidence level in finding reliable health information, and their level of trust of information obtained. Semi-structured dyadic interviews obtained information about technology, digital health, and nutrition. Transcripts were double coded by two researchers to identify nutrition service features and a >80% agreement was achieved. The mean age was 68.1±11.2 years and 65% of PwPD were male, while 80% of ICG were female. Most PwPD and ICG (82.5%) went to the internet the last time they looked up health information, and about 1/3 reported it took a lot of effort to get this information. Nearly half were concerned about the quality of the information, and approximately 70% had issues trusting health information from government agencies or diet programs. Six themes around key features for a digital nutrition intervention emerged: tailored and specific, inclusion of caregivers, promote self-efficacy, from a nutrition expert, contain a social element, and include a follow-up session. The results suggest that digital health interventions will need to be tailored to meet the needs of PwPD and their ICG.

